# Circ-ACTR2 aggravates the high glucose-induced cell dysfunction of human renal mesangial cells through mediating the miR-205-5p/HMGA2 axis in diabetic nephropathy

**DOI:** 10.1186/s13098-021-00692-x

**Published:** 2021-06-26

**Authors:** Jie Yun, Jinyu Ren, Yufei Liu, Lijuan Dai, Liqun Song, Xiaopeng Ma, Shan Luo, Yexu Song

**Affiliations:** 1 Department of Nephrology, First Affiliated Hospital, Heilongjiang University of Chinese Medicine, Harbin, China; 2 Department of Encephalopathy, Second Hospital Affiliated to Heilongjiang University of Chinese Medicine, Harbin, China; 3 Department of Blood Purification, Second Hospital Affiliated to Heilongjiang University of Chinese Medicine, Harbin, China; 4 Department of Science and Technology, Heilongjiang University of Chinese Medicine, No 26, Heping Road, Harbin, 150000 China

**Keywords:** Circ-ACTR2, Renal mesangial cells, Diabetic nephropathy, miR-205-5p, HMGA2

## Abstract

**Background:**

Circular RNAs (circRNAs) have been considered as pivotal biomarkers in Diabetic nephropathy (DN). CircRNA ARP2 actin-related protein 2 homolog (circ-ACTR2) could promote the HG-induced cell injury in DN. However, how circ-ACTR2 acts in DN is still unclear. This study aimed to explore the molecular mechanism of circ-ACTR2 in DN progression, intending to provide support for the diagnostic and therapeutic potentials of circ-ACTR2 in DN.

**Methods:**

RNA expression analysis was conducted by the quantitative reverse transcription-polymerase chain reaction (qRT-PCR). Cell growth was measured via Cell Counting Kit-8 and EdU assays. Inflammatory response was assessed by Enzyme-linked immunosorbent assay. The protein detection was performed via western blot. Oxidative stress was evaluated by the commercial kits. The molecular interaction was affirmed through dual-luciferase reporter and RNA immunoprecipitation assays.

**Results:**

Circ-ACTR2 level was upregulated in DN samples and high glucose (HG)-treated human renal mesangial cells (HRMCs). Silencing the circ-ACTR2 expression partly abolished the HG-induced cell proliferation, inflammation and extracellular matrix accumulation and oxidative stress in HRMCs. Circ-ACTR2 was confirmed as a sponge for miR-205-5p. Circ-ACTR2 regulated the effects of HG on HRMCs by targeting miR-205-5p. MiR-205-5p directly targeted high-mobility group AT-hook 2 (HMGA2), and HMGA2 downregulation also protected against cell injury in HG-treated HRMCs. HG-mediated cell dysfunction was repressed by miR-205-5p/HMGA2 axis. Moreover, circ-ACTR2 increased the expression of HMGA2 through the sponge effect on miR-205-5p in HG-treated HRMCs.

**Conclusion:**

All data have manifested that circ-ACTR2 contributed to the HG-induced DN progression in HRMCs by the mediation of miR-205-5p/HMGA2 axis.

## Introduction

Diabetic nephropathy (DN) is a serious complication resulting from diabetes mellitus (DM) in kidney, and it has become the main cause of chronic kidney disease or end-stage renal failure [1, 2]. Mesangial cells play important roles in the pathogenesis of DN [3, 4]. Mesangial cell proliferation and extracellular matrix (ECM) accumulation are related to the initiation and progression of DN [5, 6]. To explore the molecular mechanism of mesangial cell dysfunction is important for the treatment of DN. Many biological molecules have regulatory effects on DM and DN. For instance, small dense low-density lipoprotein has key function in predicting and monitoring gestational DM [7]. Glycated albumin has been considered to act as a biomarker to predict the cardiovascular risk of DM [8] and evaluate glycemic status in patients with advanced chronic kidney disease [9].

CircRNAs are non-coding closed-loop RNAs known as microRNA (miRNA) sponges and gene transcriptional or post-transcriptional regulators [10]. The specific characteristics of circRNAs such as abundant distribution, differential expression and high stability confer them the potentials as diagnostic and therapeutic biomarkers for human diseases [10]. CircRNA_010383 has been reported as a miR-135a sponge to increase the level of TRPC1 in regulating the high glucose (HG)-induced renal fibrosis in DN [11]. Circ_0000491 has exacerbated the accumulation of ECM in mesangial cells by inhibiting miR-101b to upregulate the TGFβRI expression [12].

CircRNA ARP2 actin-related protein 2 homolog (circ-ACTR2) was overexpressed in DN patients and it promoted the HG-induced cell injury in renal tubular cells [13]. However, it is unclear whether circ-ACTR2 is also correlated to the pathogenesis of DN in mesangial cells. Chen et al. have clarified that miR-205 protected against the HG-induced cell damages via targeting HMGB1 in mesangial cells and circLRP6 upregulated the HMGB1 expression through playing a sponge role of miR-205 [14]. In addition, miR-98-5p and let-7a-5p have inhibited the development of DN by downregulating the expression of high-mobility group AT-hook 2 (HMGA2) [15, 16]. The potential of circ-ACTR2 as a regulator of HMGA2 by sponging miR-205-5p remains to be investigated.

This study hypothesized that circ-ACTR2 could exert a sponge effect on miR-205-5p and HMGA2 could act as a miR-205-5p target. The circ-ACTR2/miR-205-5p/HMGA2 regulatory axis in the DN progression was studied in HG-treated renal mesangial cells.

## Materials and methods

### Human specimens

Totally, 54 kidney tissues were collected from First Affiliated Hospital, Heilongjiang University of Chinese Medicine. After the physiopathologic identification by two pathologists, these specimens have been classified into DN group (n = 27) and normal group (n = 27). The written informed consent has been acquired from each participator. Our research was authorized by the Ethics Committee of First Affiliated Hospital, Heilongjiang University of Chinese Medicine. 54 kidney tissues were frozen in liquid nitrogen for long-term preservation.

### Cell culture and treatment

Human renal mesangial cell line HRMC (BioVector NTCC Inc., Beijing, China) was maintained in cell medium produced by Dulbecco’s modified eagle medium (DMEM; Hyclone, Logan, UT, USA), 10% fetal bovine serum (FBS; Sigma-Aldrich, St. Louis, MO, USA) and 1% antibiotics (Sigma-Aldrich). Cell culture was conducted in controllable incubator (37 °C, 5% CO_2_). For the treatment of HG, cells were stimulated with 30 mM glucose (Sigma-Aldrich) for 48 h.

### Transient transfection

The specific small interfering RNA (siRNA) of circ-ACTR2 or HMGA2 (si-circ-ACTR2, si-HMGA2), miRNA mimic of miR-205-5p (miR-205-5p), and miRNA inhibitor of miR-205-5p (anti-miR-205-5p) were obtained from RIBOBIO (Guangzhou, China). The oligonucleotides si-NC, miR-NC and anti-miR-NC were applied as the corresponding negative controls. In addition, pcD5-ciR-circ-ACTR2 (circ-ACTR2) and pcDNA-HMGA2 (HMGA2) were constructed using the expression vectors pcD5-ciR (GENESEED, Guangzhou, China) and pcDNA (Invitrogen, Carlsbad, CA, USA). The operation of cell transfection was in accordance with the instruction book of Lipofectamine™ 3000 (Invitrogen) for 6 h, then cell medium was replaced with DMEM containing 30 mM glucose. After 48 h, the harvested cells were used for the subsequent experimental analysis.

### The quantitative reverse transcription-polymerase chain reaction (qRT-PCR) assay

Total RNA extraction from kidney tissues and mesangial cells was performed using TRI Reagent (Sigma-Aldrich). After the reverse transcription by Transcriptor Universal cDNA Master (Roche, Basle, Switzerland), the quantitative detection was performed by FastStart Universal SYBR Green Master (Roche) as per the user’s guideline. RNA stability was assessed by qRT-PCR after total RNA was treated with RNase R (GENESEED) at 37 °C. The subcellular localization was also analyzed using qRT-PCR after the RNA isolation from the nucleus and cytoplasm using PARIS™ Kit (Invitrogen). The 2^−∆∆Ct^ method was applied to calculate the relative expression of each molecule as previously reported [17]. Glyceraldehyde-phosphate dehydrogenase (GAPDH) and U6 were selected as the endogenetic references. The specific primers contained circ-ACTR2: 5′-ATCACGGTTGGAACGAGAAC-3′ (forward, F), 5′-TTCATGTCATCCCAATTTCG-3′ (reverse, R); ACTR2: 5′-GACTACACATTTGGACCAGAGA-3′ (F), 5′-CTTCTCTCTGTTTTTGGTTGGG-3′ (R); miR-205-5p: 5′-TCGGCAGGTCCTTCATTCCACC-3′ (F), 5′-CTCAACTGGTGTCGTGGA-3′ (R); HMGA2: 5′-CAGCAGCAAGAACCAACCG-3′ (F), 5′-TGTTGTGGCCATTTCCTAGGT-3′ (R); GAPDH: 5′-GTCTCCTCTGACTTCAACAGCG-3′ (F), 5′-ACCACCCTGTTGCTGTAGCCAA-3′ (R); U6: 5′-GCTTCGGCAGCACATATACTAAAAT-3′ (F), 5′-CGCTTCACGAATTTGCGTGTCAT-3′ (R).

### Cell counting kit-8 (CCK-8) assay

Cell viability of HRMCs was measured using Cell Counting Kit-8 (Sigma-Aldrich). 10 μL CCK-8 solution was added into each well to incubate with cells for 2 h. Then, cell viability was analyzed by detecting the optical density value of 450 nm under a microplate reader (Bio-Rad, Hercules, CA, USA).

### Enzyme-linked immunosorbent assay (ELISA)

Cell supernatants were harvested after cell treatment and transfection. ELISA kits (RayBiotech, Norcross, GA, USA) were used for the determination of interleukin-6 (IL-6) and tumor necrosis factor-alpha (TNF-α) according to the producer’s specification. The concentration was expressed as pg/mL.

### EdU assay

EdU kit (RIBOBIO) was applied to assess cell proliferative ability. Briefly, cells were incubated with EdU solution and fastened with 4% paraformaldehyde (Sigma-Aldrich). Then cells were stained with 1 × Apollo staining solution and 4′,6-diamidino-2- phenylindole (DAPI; Beyotime, Shanghai, China), followed by the cell observation on the fluorescence microscope (Olympus, Tokyo, Japan). Ultimately, the EdU-positive cells (EdU + DAPI-stained cells) were counted.

### Western blot

The detection of protein expression was conducted referring to the descriptions of issued studies [18, 19]. The primary antibodies against collagen I (ab34710, 1:1000), collagen IV (ab7536, 1:1000), HMGA2 (ab207301, 1:1000) and GAPDH (ab181602, 1:3000) were acquired from Abcam (Cambridge, UK). Goat Anti-rabbit IgG H&L (HRP) secondary antibody (Abcam, ab205718, 1:5000) was then incubated with the membranes to conjugate with the primary antibody, and then the protein blots were examined using Electrochemiluminescence Substrate Kit (Abcam). ImageJ software (NIH, Bethesda, MD, USA) was exploited for analyzing the protein level of each gene.

### Oxidative stress assay

Superoxide dismutase (SOD) activity and malondialdehyde (MDA) level were detected to evaluate oxidative stress in HRMCs. The operating procedures were performed following the guides of SOD and MDA Assay Kits (Sigma-Aldrich), followed by the respective detection of absorbance at 450 nm and 530 nm.

### Dual-luciferase reporter assay

The prediction of the targets for circ-ACTR2 and miR-205-5p was carried out by starbase v2.0 (http://starbase.sysu.edu.cn). The wild-type (WT) and mutant-type (MUT) sequences of circ-ACTR2 were respectively used to construct the WT-circ-ACTR2 and MUT-circ-ACTR2 luciferase plasmids using the pEZX-FR03 plasmid (GeneCopoeia, Rockville, MA, USA). Also, the WT-HMGA2 3′UTR and MUT-HMGA2 3′UTR plasmids were generated. Plasmid transfection was performed in HRMCs together with miR-NC or miR-205-5p. After 48 h, the luciferase signal of each group was determined via the Luc-Pair™ Duo-Luciferase HS Assay Kit (GeneCopoeia).

### RNA immunoprecipitation (RIP) assay

The target binding was also analyzed using RNA Immunoprecipitation Kit (Sigma-Aldrich). In brief, the magnetic beads coated with antibody of Argonaute-2 (Ago2) or immunoglobulin G (IgG) were respectively incubated to the harvested cells. Subsequently, the RNA complexes binding to the magnetic beads were isolated and the qRT-PCR was conducted to quantify the molecular expression (circ-ACTR2, miR-205-5p and HMGA2).

### Statistical analysis

Statistical analysis in the current study was completed using SPSS 22.0 (SPSS Inc., Chicago, IL, USA). After three independent assays, data have been expressed as the mean ± standard deviation (SD). For the linear analysis in human samples, Pearson’s correlation coefficient was applied to analyze the expression levels between two targets. Student’s *t*-test for two groups and one-way analysis of variance (ANOVA) followed by Tukey’s test for multiple groups were adopted to assess the statistical difference. *P* < 0.05 indicated that the difference was conspicuous.

## Results

### The high expression of circ-ACTR2 was detected in DN samples and HG-treated HRMCs

**Fig. 1:**
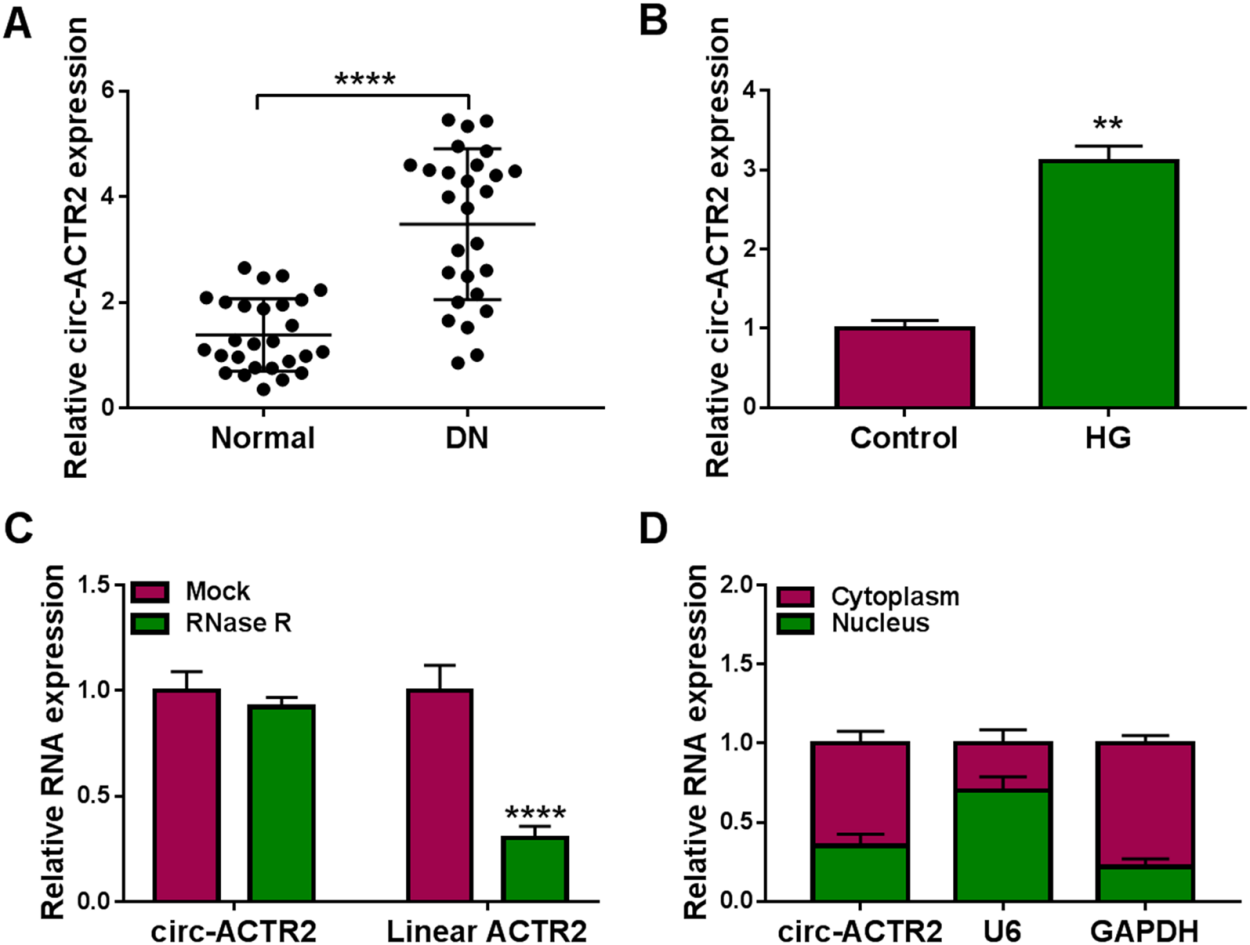
The high expression of circ-ACTR2 was detected in DN samples and HG-treated HRMCs. **A**, **B** The qRT-PCR was performed to detect the circ-ACTR2 level in normal and DN samples (**A**) or control and HG-treated HRMCs (**B**). **C** The determination of circ-ACTR2 and linear ACTR2 was conducted by qRT-PCR after treatment of RNase R in total RNA. **D** The circ-ACTR2, U6 and GAPDH levels were assayed by qRT-PCR in cytoplasm and nucleus. **P < 0.01, ****P < 0.0001

The expression pattern of circ-ACTR2 was examined by qRT-PCR in tissue samples and cells. Relative to the 27 normal samples, circ-ACTR2 was highly expressed in 27 DN samples (Fig. 1A). HG treatment also induced the upregulation of circ-ACTR2 in HRMCs contrasted with the control group (Fig. 1B). The stability analysis revealed that circ-ACTR2 have higher resistance to RNase R than linear ACTR2 (Fig. 1C). The qRT-PCR detection in cytoplasm and nucleus demonstrated that circ-ACTR2 was mainly localized in the cytoplasm of HRMCs (Fig. 1D). It has been identified that circ-ACTR2 was differentially expressed in DN.

### HG-induced cell damages in HRMCs were partly reversed by the silence of circ-ACTR2

**Fig. 2:**
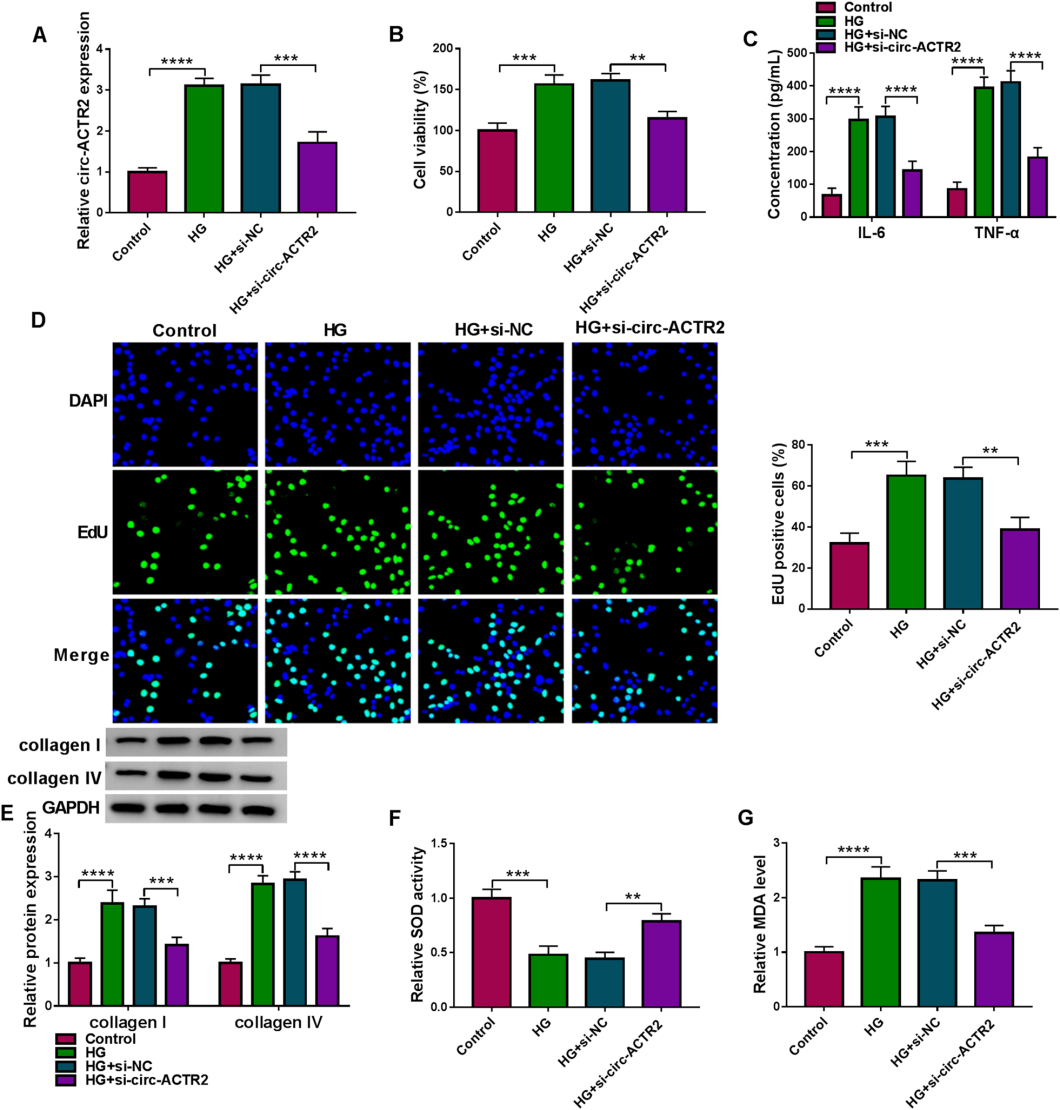
HG-induced cell damages in HRMCs were partly reversed by the silence of circ-ACTR2. **A** The expression of circ-ACTR2 was quantified using qRT-PCR in four groups of control, HG, HG + si-NC, HG + si-ACTR2 in HRMCs. **B** Cell viability detection was performed by CCK-8 assay. **C** Inflammatory cytokines IL-6 and TNF-α were determined by ELISA. **D** Cell proliferation analysis was performed by EdU assay. **E** The protein examination of collagen I and collagen IV was conducted by western blot. **F**, **G** SOD activity (**F**) and MDA level (**G**) were respectively measured using the corresponding kits. **P < 0.01, ***P < 0.001, ****P < 0.0001

The silence of circ-ACTR2 was achieved by the transfection of siRNA targeting circ-ACTR2. As shown in Fig. 2A, si-circ-ACTR2 transfection inhibited the HG-mediated upregulation of circ-ACTR2 expression. The treatment of HG was displayed to enhance cell viability, which was then abated by si-circ-ACTR2 (Fig. 2B). ELISA manifested that the promoting effects of HG on IL-6 and TNF-α were attenuated following the downregulation of circ-ACTR2 level (Fig. 2C). According to the data of EdU assay, cell proliferation was also restored to a normal level after the si-circ-ACTR2 transfection in HG-treated HRMCs (Fig. 2D). Western blot results indicated that collagen I and collagen IV protein levels were lower in HG + si-circ-ACTR2 group than these in HG + si-NC group, suggesting that knockdown of circ-ACTR2 alleviated the HG-induced ECM deposition (Fig. 2E). SOD activity was inhibited (Fig. 2F) and MDA level was increased (Fig. 2G) in HG-treated HRMCs, while the oxidative stress mediated by HG was counteracted with the expression inhibition of circ-ACTR2. All in all, silencing the expression of circ-ACTR2 partly abolished the HG-induced cell damages in HRMCs.

### Circ-ACTR2 exhibited the sponge function of miR-205-5p

**Fig. 3:**
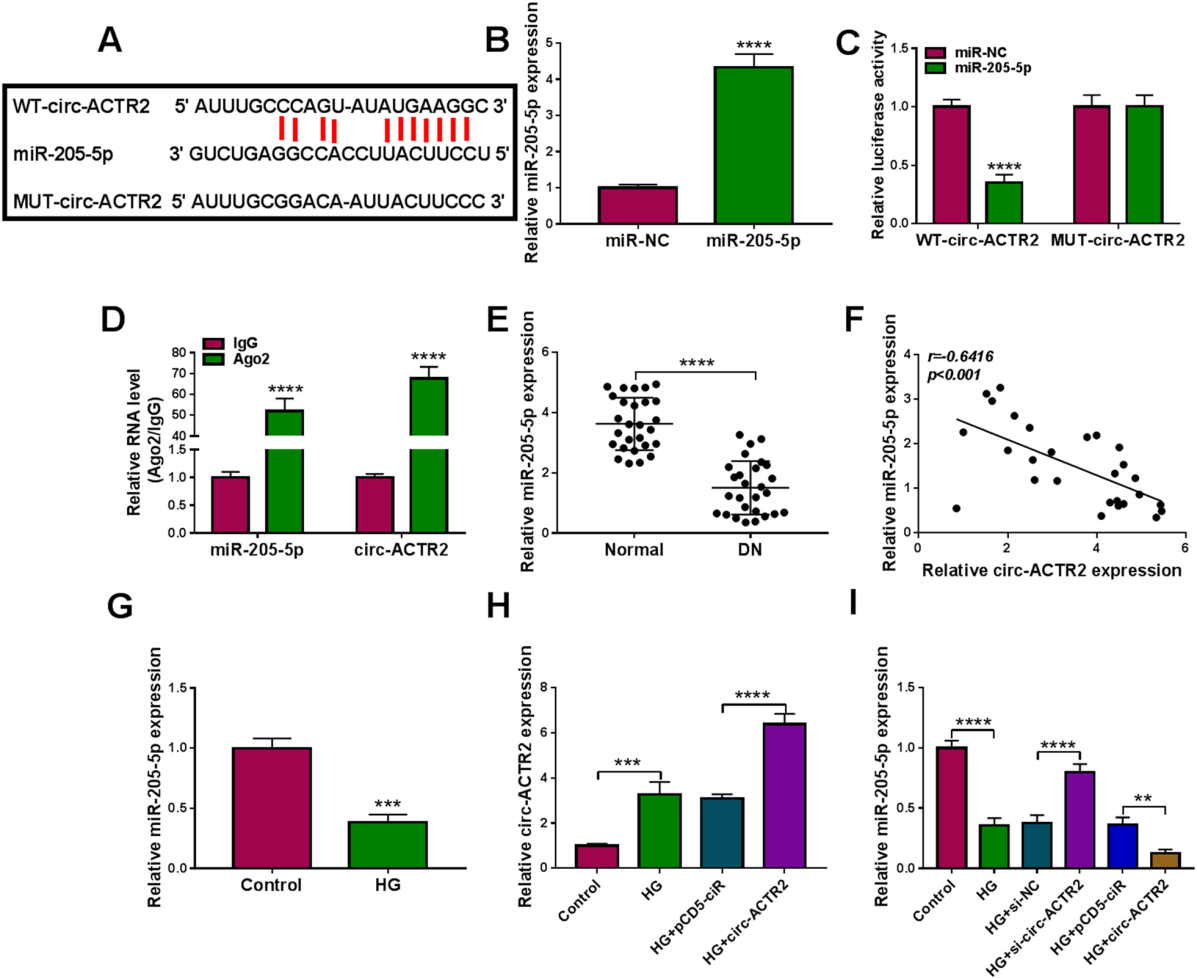
Circ-ACTR2 exhibited the sponge function of miR-205-5p. **A** Starbase v2.0 predicted the binding sites between circ-ACTR2 and miR-205-5p. **B** The miR-205-5p level was examined by qRT-PCR after transfection of miR-NC or miR-205-5p. **C**, **D** Dual-luciferase reporter assay (**C**) and RIP assay (**D**) were used to confirm whether circ-ACTR2 combined with miR-205-5p. **E** The quantification of miR-205-5p was carried out using qRT-PCR in normal and DN tissues. **F** The linear analysis between circ-ACTR2 and miR-205-5p was performed using the Pearson’s correlation coefficient. **G** The effect of HG on the miR-205-5p expression was analyzed via qRT-PCR. **H** The level of circ-ACTR2 was detected by qRT-PCR in control, HG, HG + pcD5-ciR or HG + circ-ACTR2 group. **I** The regulation of circ-ACTR2 inhibition or overexpression on the miR-205-5p expression was performed by qRT-PCR in HG-treated HRMCs. **P < 0.01, ***P < 0.001, ****P < 0.0001

The online prediction by starbase v2.0 identified that circ-ACTR2 had the target binding sites of miR-205-5p (Fig. 3A). In comparison to the miR-NC group, the miR-205-5p expression was elevated by fourfold changes in the miR-205-5p group (Fig. 3B). Then, the overexpression of miR-205-5p was found to decrease the relative luciferase activity of WT-circ-ACTR2 group instead of MUT-circ-ACTR2 group (Fig. 3C). Meanwhile, miR-205-5p and circ-ACTR2 levels were higher in Ago2 group than these in IgG group of RIP assay (Fig. 3D). The expression analysis for miR-205-5p displayed that it was downregulated in DN samples by contrast to normal samples (Fig. 3E). Interestingly, a negative correlation (*r* = − 0.6416, *p* < 0.001) was noticed between the expression levels of miR-205-5p and circ-ACTR2 in DN samples (Fig. 3F). In addition, the level of miR-205-5p was signally declined in HG group compared to the control group in HRMCs (Fig. 3G). The transfection of circ-ACTR2 further enhanced the upregulation of circ-ACTR2 caused by HG, indicating that the transfection efficiency of circ-ACTR2 was excellent (Fig. 3H). HG-mediated inhibitory effect on the miR-205-5p expression was promoted by the knockdown of circ-ACTR2 but this effect was repressed by the overexpression of circ-ACTR2 (Fig. 3I). These data testified that circ-ACTR2 served as a miR-205-5p sponge.

### Circ-ACTR2/miR-205-5p axis affected the regulation of HG in HRMCs

**Fig. 4:**
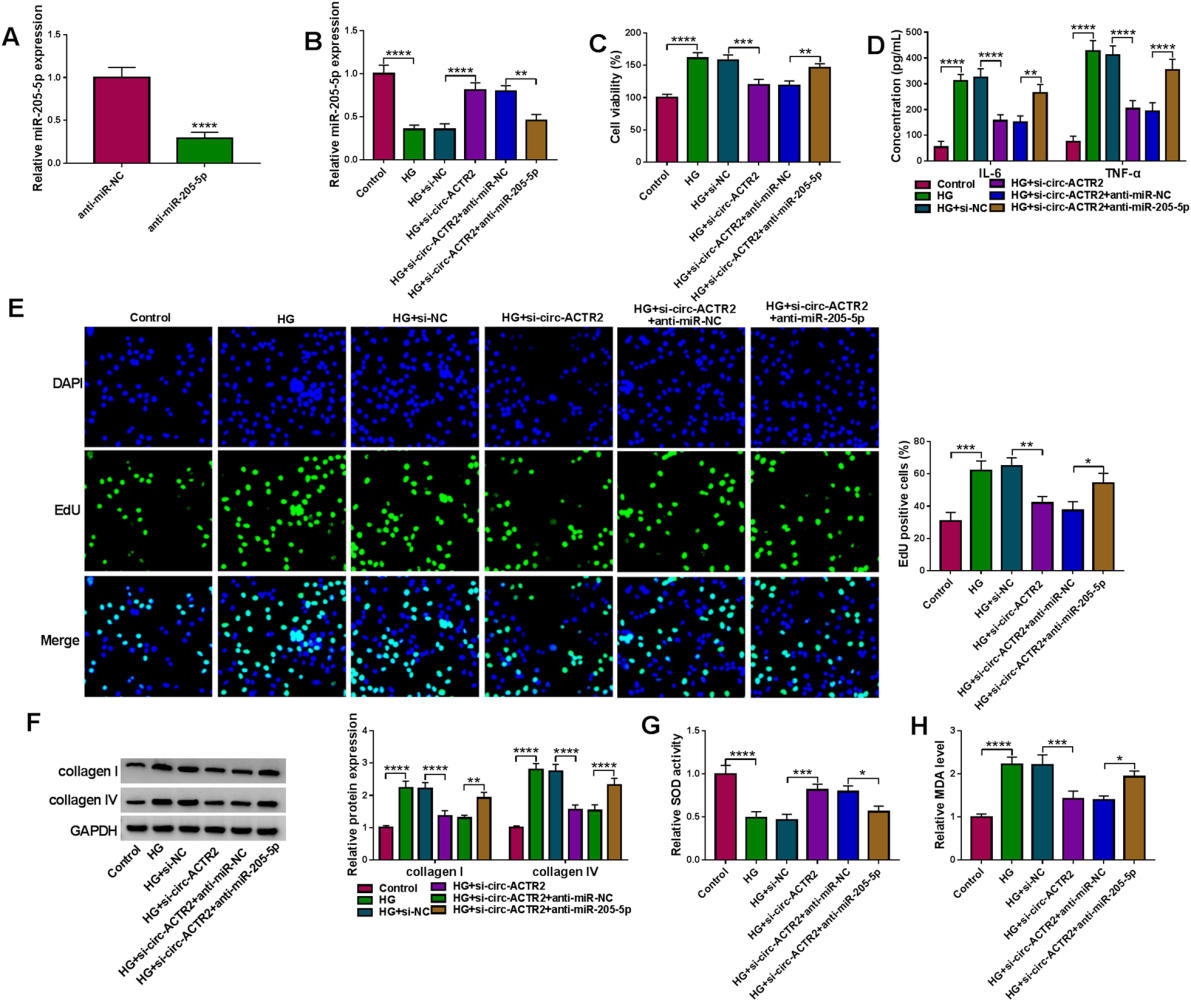
Circ-ACTR2/miR-205-5p axis affected the regulation of HG in HRMCs. **A** The transfection efficiency of anti-miR-205-5p was assessed by qRT-PCR. **B** The qRT-PCR was applied for the expression detection of miR-205-5p after transfection of si-NC, si-ACTR2, si-ACTR2 + anti-miR-NC or si-ACTR2 + anti-miR-205-5p in HG-treated HRMCs. **C** Cell viability was examined via CCK-8 assay. **D** The concentrations of IL-6 and TNF-α were tested via ELSIA. **E** Cell proliferation was assessed via EdU assay. **F**, **G** The levels of collagen I and collagen IV were assayed via western blot. **H**, **I** Oxidative stress was evaluated by SOD activity (**H**) and MDA level (**I**) using the kits. *P < 0.05, **P < 0.01, ***P < 0.001, ****P < 0.0001

The inhibitory efficiency of anti-miR-205-5p was significant in HRMCs relative to anti-miR-NC group (Fig. 4A). In addition, anti-miR-205-5p also eliminated the si-circ-ACTR2-induced overexpression of miR-205-5p in HG-treated HRMCs (Fig. 4B). Furthermore, the suppressive effects of circ-ACTR2 knockdown on cell viability (Fig. 4C), inflammatory response (Fig. 4D) and cell proliferation (Fig. 4E) in HG-treated HRMCs were mitigated by anti-miR-205-5p. The downregulation of miR-205-5p also abrogated the si-circ-ACTR2-mediated inhibition of ECM accumulation (Fig. 4F, G) and oxidative stress (Fig. 4H, I). The above evidence showed that circ-ACTR2 regulated cell injury in HG-treated HRMCs by targeting miR-205-5p.

### HMGA2 was found as a downstream target of miR-205-5p

**Fig. 5:**
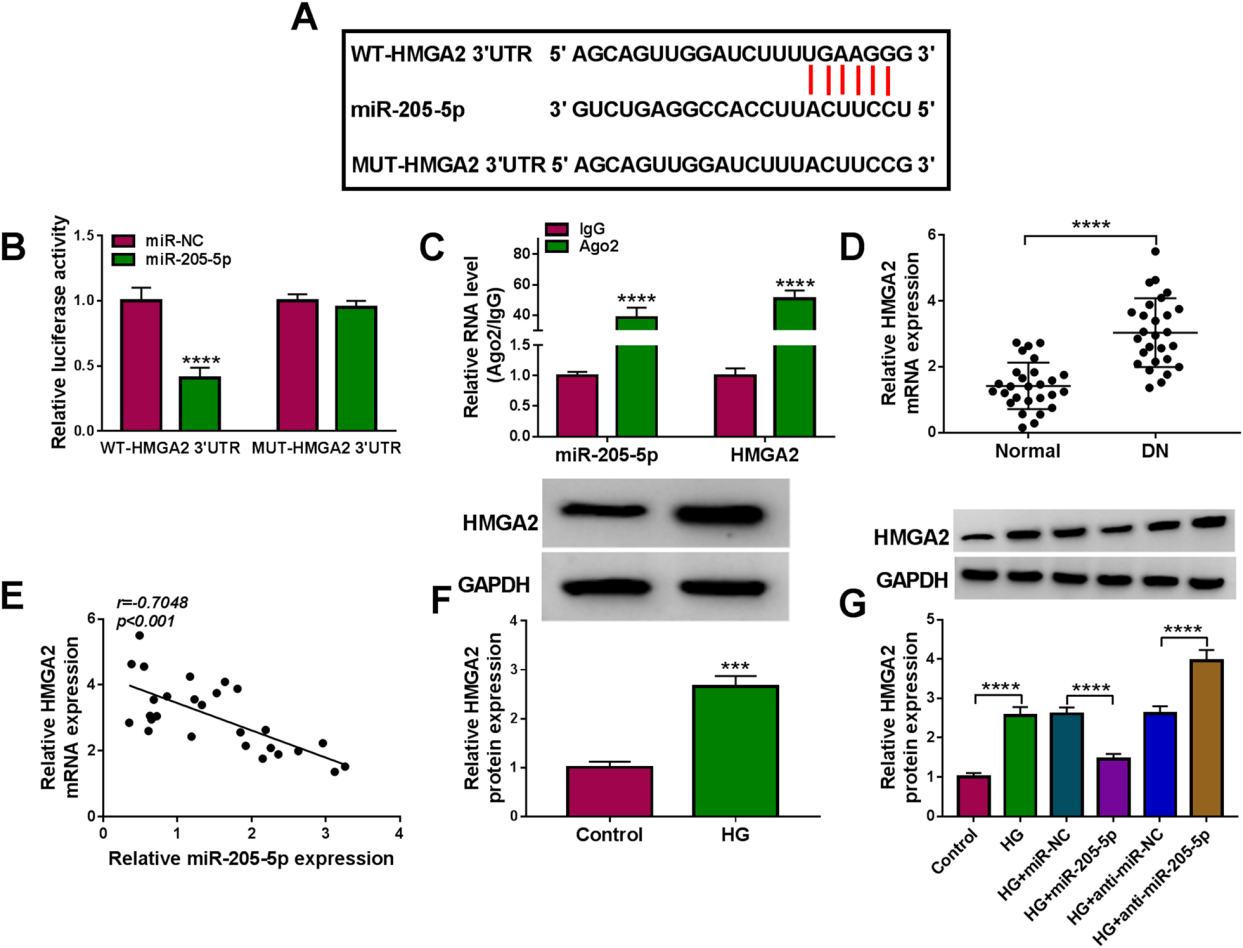
HMGA2 was found as a downstream target of miR-205-5p. **A** The binding sites of miR-205-5p in HMGA2 3′UTR was predicted by starbase v2.0. **B**, **C** The binding between miR-205-5p and HMGA2 was explored using dual-luciferase reporter assay (**B**) and RIP assay (**C**). **D** HMGA2 mRNA expression was assayed by qRT-PCR in normal and DN samples. **E** The linear relation between the expression of miR-205-5p and HMGA2 was analyzed by Pearson’s correlation coefficient. **F** The protein detection of HMGA2 was performed by western blot in control and HG groups. **G** Western blot was used to assess the effect of miR-205-5p on the HMGA2 protein level. ***P < 0.001, ****P < 0.0001

The binding sites between the 3′UTR sequence of HMGA2 and miR-205-5p sequence were also shown in starbase v2.0 (Fig. 5A). Dual-luciferase reporter assay (Fig. 5B) and RIP assay (Fig. 5C) further affirmed the interaction between miR-205-5p and HMGA2. HMGA2 mRNA expression was higher in DN tissues than that in normal tissues (Fig. 5D) and HMGA2 was negatively related to the miR-205-5p expression (*r* = − 0.7048, *p* < 0.001) in DN tissues (Fig. 5E). The protein level of HMGA2 was increased after the treatment of HG in HRMCs (Fig. 5F). Moreover, miR-205-5p overexpression inhibited the stimulative effect of HG on the HMGA2 protein expression but miR-205-5p downregulation aggravated this effect (Fig. 5G). Altogether, miR-205-5p could directly target HMGA2.

### HMGA2 downregulation protected against the HG-induced cell dysfunction in HRMCs

**Fig. 6:**
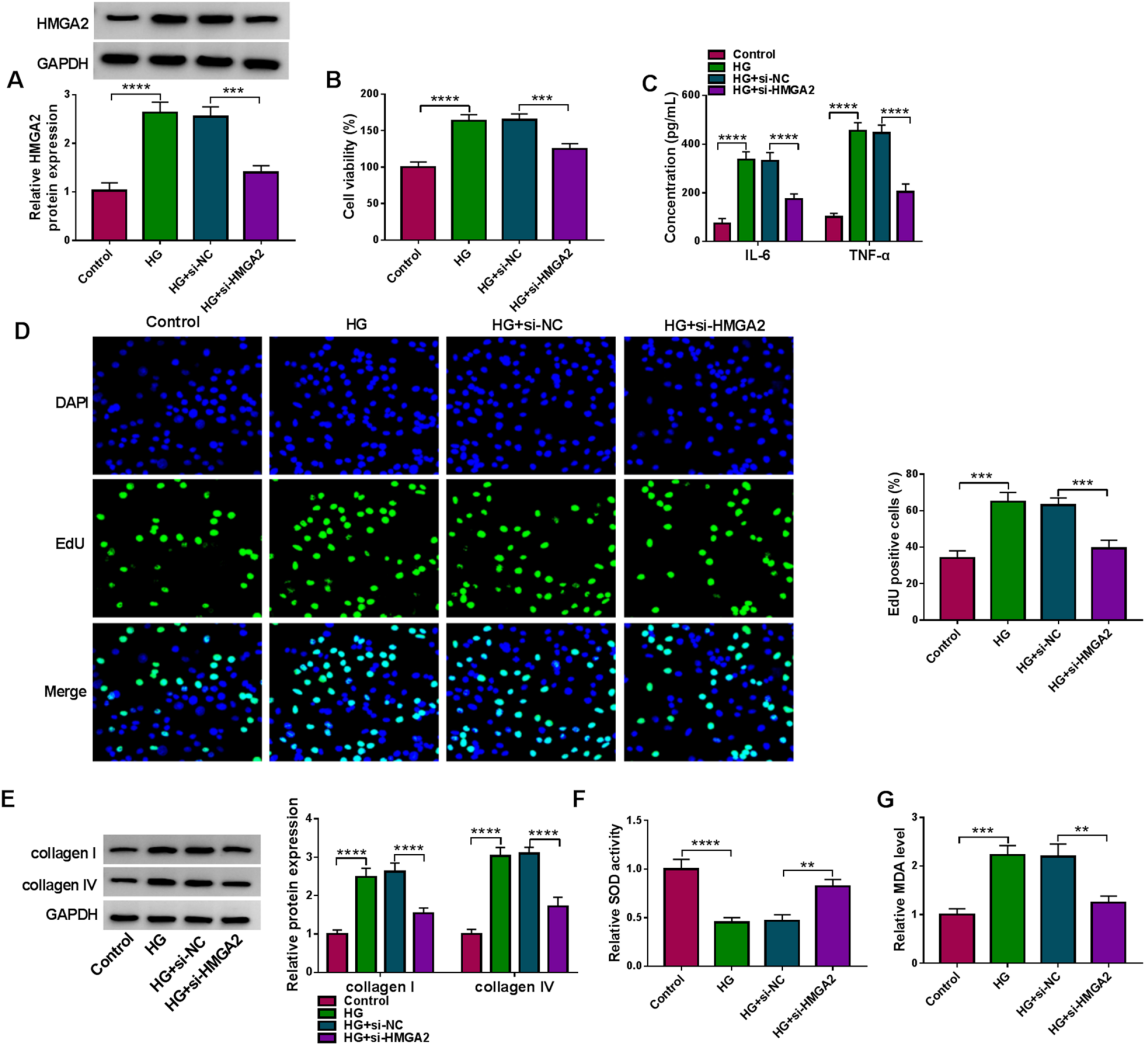
HMGA2 downregulation protected against the HG-induced cell dysfunction in HRMCs. **A** The protein expression of HMGA2 in control, HG, HG + si-NC or HG + si-HMGA2 group was evaluated by western blot. **B** CCK-8 was performed to analyze cell viability. **C** ELISA was performed to measure the concentrations of IL-6 and TNF-α. **D** EdU assay was conducted to determine cell proliferation. **E**, **F** Western blot was conducted to detect the protein levels of collagen I and collagen IV. **G**, **H** SOD activity (**G**) and MDA level (**H**) by the detection kits were exploited to examine the oxidative stress. **P < 0.01, ***P < 0.001, ****P < 0.0001

The function of HMGA2 in DN was also explored using the loss-of-function method. Western blot showed that HMGA2 protein level was inhibited in HG + si-HMGA2 group contrasted to HG + si-NC group (Fig. 6A). Subsequent assays demonstrated that cell viability (Fig. 6B), inflammatory IL-6/TNF-α levels (Fig. 6C) and cell proliferation (Fig. 6D) in HG-treated HRMCs were all suppressed by the introduction of si-HMGA2. The protein levels of collagen I and collagen IV were also suppressed by si-HMGA2 under the treatment of HG (Fig. 6E, F). Additionally, the HG-triggered repression of SOD activity (Fig. 6G) and promotion of MDA level (Fig. 6H) were reverted after the HMGA2 expression was downregulated. The knockdown of HMGA2 weakened the HG-induced cell growth, inflammation, ECM deposition and oxidative stress.

### MiR-205-5p/HMGA2 axis was involved in the regulation of HG-induced DN progression

**Fig. 7:**
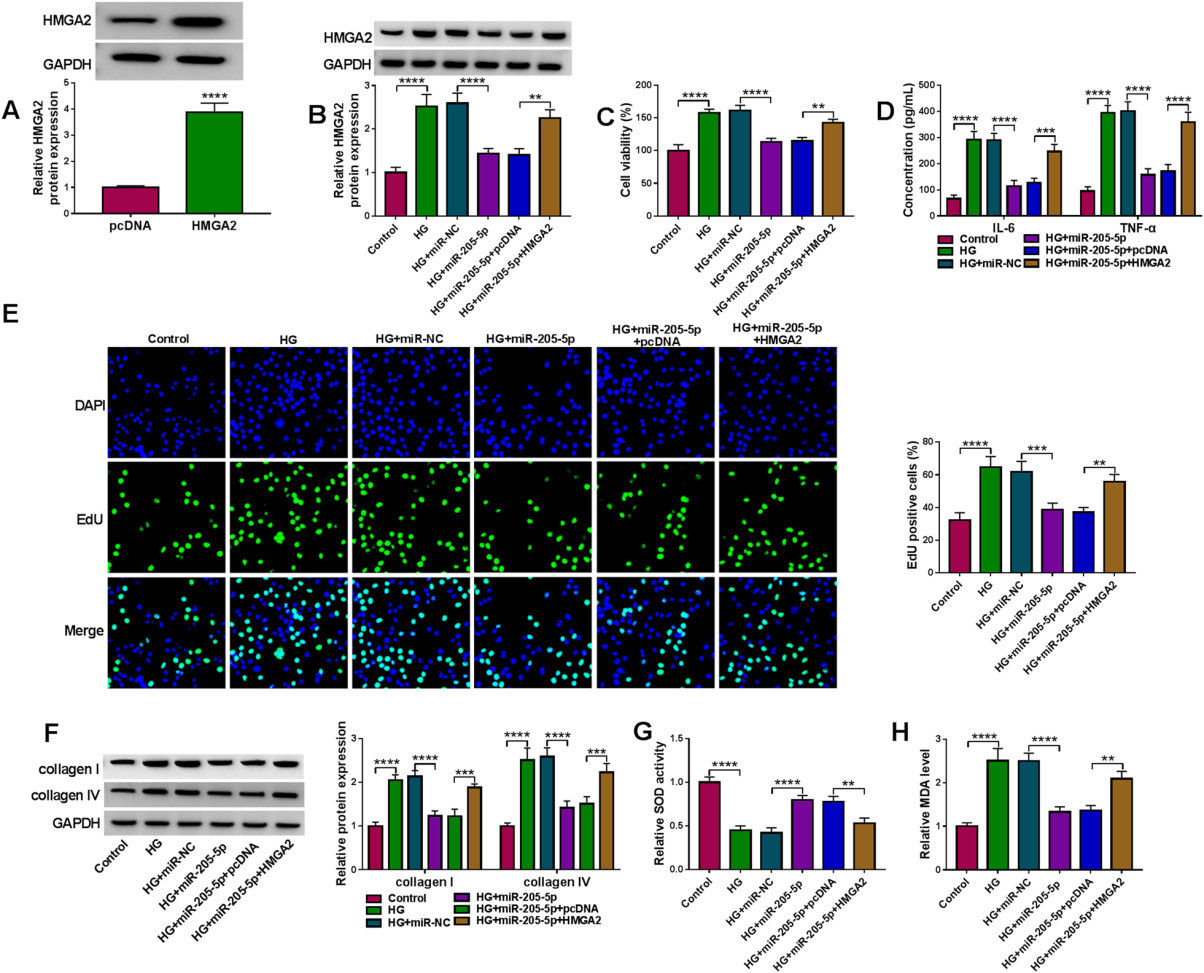
MiR-205-5p/HMGA2 axis was involved in the regulation of HG-induced DN progression. **A** The overexpression effect of HMGA2 transfection on the HMGA2 protein expression was analyzed using western blot. **B** HMGA2 protein detection was performed by western blot in control, HG, HG + miR-NC, HG + miR-205-5p, HG + miR-205-5p + pcDNA or HG + miR-205-5p + HMGA2 group. **C** CCK-8 was used for the examination of cell viability. **D** ELISA was used for the determination of IL-6 and TNF-α. **E** EdU assay was used for the analysis of cell proliferation. **F**, **G** Western blot was applied for the protein measurement of collagen I and collagen IV. **H**, **I** SOD activity (**G**) and MDA level (**H**) by the respective kits were applied for the assessment of oxidative stress. **P < 0.01, ***P < 0.001, ****P < 0.0001

HMGA2 protein expression was increased by transfection of HMGA2 with more than threefold changes (Fig. 7A). The repressive regulation of miR-205-5p on the HMGA2 protein level was offset after the HMGA2 transfection (Fig. 7B). The overexpression of miR-205-5p returned the HG-aroused promotion of cell viability (Fig. 7C), inflammatory response (Fig. 7D) and cell proliferation (Fig. 7E), whereas the upregulation of HMGA2 restrained the reversal of miR-205-5p. As the results of HMGA2 overexpression, the inhibitory influences of miR-205-5p on ECM aggregation (Fig. 7F, G) and oxidative stress (Fig. 7H, I) were counterbalanced in HG-treated HRMCs. Overall, miR-205-5p targeted HMGA2 to impede the HG-induced DN progression.

### Circ-ACTR2 regulated the HMGA2 level by acting as a sponge of miR-205-5p

**Fig. 8:**
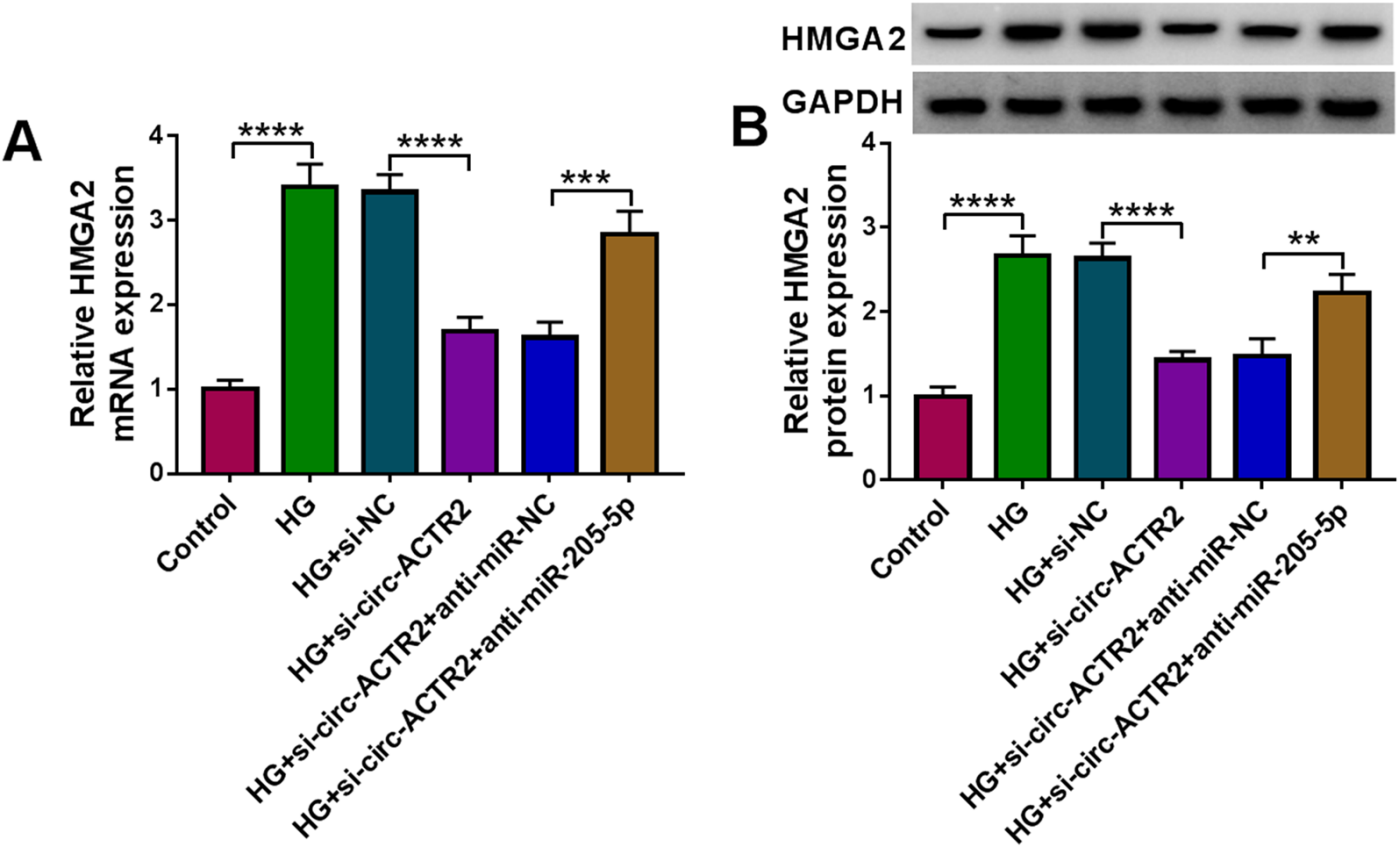
Circ-ACTR2 regulated the HMGA2 level by acting as a sponge of miR-205-5p. **A**, **B** The mRNA (**A**) and protein (**B**) levels of HMGA2 were respectively detected by qRT-PCR and western blot after transfection of si-NC, si-circ-ACTR2, si-circ-ACTR2 + anti-miR-NC or si-circ-ACTR2 + anti-miR-205-5p in HG-treated HRMCs. **P < 0.01, ****P < 0.0001

The effect of circ-ACTR2 on the expression of HMGA2 was investigated. As depicted in Fig. 8A, B, the mRNA and protein levels of HMGA2 in HG-treated HRMCs were downregulated by si-circ-ACTR2 while this downregulation was countervailed by miR-205-5p inhibitor. Thus, circ-ACTR2 could increase the expression level of HMGA2 by targeting miR-205-5p.

## Discussion

Circ-ACTR2 has been identified to participate in the HG-induced mesangial cell dysfunction by modulating the miR-205-5p and HMGA2 levels, which confirmed that circ-ACTR2/miR-205-5p/HMGA2 axis was present in the DN progression.

A large amount of evidence has shown that circRNAs regulated the pathological processes of renal diseases [20]. For example, circSDHC contributed to cell invasion and proliferation in renal cell carcinoma [21]; circ-Ttc3 promoted the inflammatory response and oxidative stress in sepsis-induced acute kidney injury [22]; hsa_circ_0123190 was significantly downregulated in lupus nephritis and it might impede the progression of lupus nephritis [23]. Circ-ACTR2 has been upregulated in DN patients and HG-treated renal tubular cells [13]. Consistently, the high expression of circ-ACTR2 was also confirmed in our DN samples and HG-stimulated renal mesangial cells. Our assays further demonstrated that the downregulation of circ-ACTR2 antagonized the HG-induced cell proliferation, ECM accumulation, inflammatory and oxidative damages. These results implied that circ-ACTR2 improved the developing process of DN in vitro, in accordance with the previous findings [13].

CircRNAs played the regulatory roles by functioning as the natural sponges of miRNAs [24]. MiR-205-5p was considered as a candidate miRNA target of circ-ACTR2 using starbase v2.0. The issued studies have manifested the sponge effects of some circRNAs on miR-205-5p. Circ_NOTCH3 served as a proto-oncogene in basal-like breast carcinoma by sponging miR-205-5p [25], and circPVT1 improved the malignant development of osteosarcoma through the sponge mechanism of miR-205-5p [26]. Cao et al. declared that circ0001429 accelerated cell propagation and invasiveness in bladder cancer by interacting with miR-205-5p [27]. This study verified that circ-ACTR2 could sponge miR-205-5p in HG-treated HRMCs and its function in the DN progression was associated with the regulation of miR-205-5p expression.

MiRNAs also affect the developing processes of diseases via silencing the downstream targets. We have validated that miR-205-5p targeted HMGA2 in the current research. HMGA2 has been revealed to increase the risk of developing nephropathy in those patients with type 2 diabetes [28]. Also, our functional analysis suggested that HMGA2 could enhance the HG-mediated cell injury in HRMCs. Furthermore, the protective role of miR-205-5p in HG-induced DN was achieved by reducing the level of HMGA2.

CircRNAs are implicated in the development of various diseases by targeting miRNAs to generate the expression changes of genes. CircZFR exerted the oncogenic function in hepatocellular carcinoma by affecting the miR-375-mediated HMGA2 expression [29]. CircRNA_000203 promoted cardiac hypertrophy by inhibiting miR-26b-5p and miR-140-3p to enhance the Gata4 level [30]. Increasing circRNA/miRNA/mRNA axes have been discovered in the regulation of various diabetic complications [31–33]. In this report, we found that circ-ACTR2 evoked the overexpression of HMGA2 by targeting miR-205-5p in HG-induced DN.

## Conclusion

In conclusion, circ-ACTR2 was affirmed to facilitate the HG-mediated DN progression in mesangial cells by the miR-205-5p/HMGA2 axis. This signal mechanism enhanced the pathogenic understanding of mesangial cells in DN. Circ-ACTR2 is promising to be a molecular target to improve the diagnosis and treatment of DN.

## Data Availability

Not applicable.

## References

[1] Eftekhari A, Vahed SZ, Kavetskyy T (2020). Cell junction proteins: crossing the glomerular filtration barrier in diabetic nephropathy. Int J Biol Macromol.

[2] Xiong Y, Zhou L (2019). The signaling of cellular senescence in diabetic nephropathy. Oxid Med Cell Longev.

[3] Tung CW, Hsu YC, Shih YH (2018). Glomerular mesangial cell and podocyte injuries in diabetic nephropathy. Nephrology.

[4] Wakisaka M, Kamouchi M, Kitazono T (2019). Lessons from the trials for the desirable effects of sodium glucose co-transporter 2 inhibitors on diabetic cardiovascular events and renal dysfunction. Int J Mol Sci.

[5] Deng Y, Lan T, Huang J (2014). Sphingosine kinase-1/sphingosine 1-phosphate pathway in diabetic nephropathy. Chin Med J.

[6] Gnudi L (2016). Angiopoietins and diabetic nephropathy. Diabetologia.

[7] Rizvi AA, Cuadra S, Nikolic D (2014). Gestational diabetes and the metabolic syndrome: can obesity and small, dense low density lipoproteins be key mediators of this association?. Curr Pharm Biotechnol.

[8] Giglio RV, Lo Sasso B, Agnello L (2020). Recent updates and advances in the use of glycated albumin for the diagnosis and monitoring of diabetes and renal, cerebro- and cardio-metabolic diseases. J Clin Med.

[9] Bellia C, Cosma C, Lo Sasso B (2019). Glycated albumin as a glycaemic marker in patients with advanced chronic kidney disease and anaemia: a preliminary report. Scand J Clin Lab Invest.

[10] Zhang L, Zhang Y, Wang Y (2020). Circular RNAs: functions and clinical significance in cardiovascular disease. Front Cell Dev Biol.

[11] Peng F, Gong W, Li S (2020). circRNA_010383 acts as a sponge for miR-135a and its downregulated expression contributes to renal fibrosis in diabetic nephropathy. Diabetes.

[12] Mou X, Chenv JW, Zhou DY (2020). A novel identified circular RNA, circ_0000491, aggravates the extracellular matrix of diabetic nephropathy glomerular mesangial cells through suppressing miR101b by targeting TGFbetaRI. Mol Med Rep.

[13] Wen S, Li S, Li L (2020). circACTR2: a novel mechanism regulating high glucose-induced fibrosis in renal tubular cells via pyroptosis. Biol Pharm Bull.

[14] Chen B, Li Y, Liu Y (2019). circLRP6 regulates high glucose-induced proliferation, oxidative stress, ECM accumulation, and inflammation in mesangial cells. J Cell Physiol.

[15] Zhu Y, Xu J, Liang W (2019). miR-98-5p alleviated epithelial-to-mesenchymal transition and renal fibrosis via targeting Hmga2 in diabetic nephropathy. Int J Endocrinol.

[16] Wang T, Zhu H, Yang S (2019). Let7a5p may participate in the pathogenesis of diabetic nephropathy through targeting HMGA2. Mol Med Rep.

[17] Livak KJ, Schmittgen TD (2001). Analysis of relative gene expression data using real-time quantitative PCR and the 2(-Delta Delta C(T)) method. Methods.

[18] Ogoyama M, Ohkuchi A, Takahashi H (2021). LncRNA H19-derived miR-675-5p accelerates the invasion of extravillous trophoblast cells by inhibiting GATA2 and subsequently activating matrix metalloproteinases. Int J Mol Sci.

[19] Teng F, Zhang JX, Chen Y (2021). LncRNA NKX2-1-AS1 promotes tumor progression and angiogenesis via upregulation of SERPINE1 expression and activation of the VEGFR-2 signaling pathway in gastric cancer. Mol Oncol.

[20] Jin J, Sun H, Shi C (2020). Circular RNA in renal diseases. J Cell Mol Med.

[21] Cen J, Liang Y, Huang Y (2021). Circular RNA circSDHC serves as a sponge for miR-127-3p to promote the proliferation and metastasis of renal cell carcinoma via the CDKN3/E2F1 axis. Mol Cancer.

[22] Ma X, Zhu G, Jiao T (2021). Effects of circular RNA Ttc3/miR-148a/Rcan2 axis on inflammation and oxidative stress in rats with acute kidney injury induced by sepsis. Life Sci.

[23] Zhang C, Gao C, Di X (2021). Hsa_circ_0123190 acts as a competitive endogenous RNA to regulate APLNR expression by sponging hsa-miR-483-3p in lupus nephritis. Arthritis Res Ther.

[24] Tang Q, Hann SS (2020). Biological roles and mechanisms of circular RNA in human cancers. Onco Targets Ther.

[25] Guan B, Li Q, Zhang HZ (2020). circ_NOTCH3 functions as a protooncogene competing with miR-205-5p, modulating KLF12 expression and promoting the development and progression of basal-like breast carcinoma. Front Oncol.

[26] Liu YP, Wan J, Long F (2020). circPVT1 facilitates invasion and metastasis by regulating miR-205-5p/c-FLIP axis in osteosarcoma. Cancer Manag Res.

[27] Cao W, Zhao Y, Wang L (2019). Circ0001429 regulates progression of bladder cancer through binding miR-205-5p and promoting VEGFA expression. Cancer Biomark.

[28] Alkayyali S, Lajer M, Deshmukh H (2013). Common variant in the HMGA2 gene increases susceptibility to nephropathy in patients with type 2 diabetes. Diabetologia.

[29] Xu R, Yin S, Zheng M (2021). Circular RNA circZFR promotes hepatocellular carcinoma progression by regulating miR-375/HMGA2 axis. Dig Dis Sci.

[30] Li H, Xu JD, Fang XH (2020). Circular RNA circRNA_000203 aggravates cardiac hypertrophy via suppressing miR-26b-5p and miR-140-3p binding to Gata4. Cardiovasc Res.

[31] Wang Q, Cang Z, Shen L (2021). circ_0037128/miR-17-3p/AKT3 axis promotes the development of diabetic nephropathy. Gene.

[32] Liao S, Lin X, Mo C (2020). Integrated analysis of circRNA-miRNA-mRNA regulatory network identifies potential diagnostic biomarkers in diabetic foot ulcer. Noncoding RNA Res.

[33] Wu Z, Liu B, Ma Y (2020). Discovery and validation of hsa_circ_0001953 as a potential biomarker for proliferative diabetic retinopathy in human blood. Acta Ophthalmol.

